# Unravelling Soil Fungal Communities from Different Mediterranean Land-Use Backgrounds

**DOI:** 10.1371/journal.pone.0034847

**Published:** 2012-04-20

**Authors:** Alberto Orgiazzi, Erica Lumini, R. Henrik Nilsson, Mariangela Girlanda, Alfredo Vizzini, Paola Bonfante, Valeria Bianciotto

**Affiliations:** 1 Department of Life Science and Systems Biology, University of Turin, Turin, Italy; 2 Plant Protection Institute - Turin UOS, National Research Council (CNR), Turin, Italy; 3 Department of Biological and Environmental Sciences, University of Gothenburg, Gothenburg, Sweden; University of Tartu, Estonia

## Abstract

**Background:**

Fungi strongly influence ecosystem structure and functioning, playing a key role in many ecological services as decomposers, plant mutualists and pathogens. The Mediterranean area is a biodiversity hotspot that is increasingly threatened by intense land use. Therefore, to achieve a balance between conservation and human development, a better understanding of the impact of land use on the underlying fungal communities is needed.

**Methodology/Principal Findings:**

We used parallel pyrosequencing of the nuclear ribosomal ITS regions to characterize the fungal communities in five soils subjected to different anthropogenic impact in a typical Mediterranean landscape: a natural cork-oak forest, a pasture, a managed meadow, and two vineyards. Marked differences in the distribution of taxon assemblages among the different sites and communities were found. Data analyses consistently indicated a sharp distinction of the fungal community of the cork oak forest soil from those described in the other soils. Each soil showed features of the fungal assemblages retrieved which can be easily related to the above-ground settings: ectomycorrhizal phylotypes were numerous in natural sites covered by trees, but were nearly completely missing from the anthropogenic and grass-covered sites; similarly, coprophilous fungi were common in grazed sites.

**Conclusions/Significance:**

Data suggest that investigation on the below-ground fungal community may provide useful elements on the above-ground features such as vegetation coverage and agronomic procedures, allowing to assess the cost of anthropogenic land use to hidden diversity in soil. Datasets provided in this study may contribute to future searches for fungal bio-indicators as biodiversity markers of a specific site or a land-use degree.

## Introduction

The earth’s habitats and biotas are undergoing dramatic loss and biological impoverishment [1]. Human actions are causing a biodiversity crisis, with species extinction rates up to 1000 times higher than background [2]. Thus, conservation strategies represent a crucial issue especially in the Mediterranean biome because this area, covering a mere 2% of the Earth’s land surface, houses 20% of the world’s total floristic richness [3] and represents one of the smallest and most vulnerable of the Earth’s thirteen terrestrial biomes [4].

The Mediterranean Basin is recognized as one of the priority regions for conservation in Europe and has been identified as one of the 25 most important biodiversity hotspots on Earth [5]. Achieving a balance between biodiversity conservation and human development is critical for the Mediterranean area. In this context, for example, the conversion of typical cork-oak (*Quercus suber* L.) woodlands to pasture or crop land can impair soil biodiversity and functioning, with negative consequences on ecosystem service delivery, primary production, and soil sustainability [6]. The design and implementation of sustainable soil management strategies therefore require a thorough knowledge of soil biodiversity [7].

Soils represent a huge reservoir of biodiversity with several billion prokaryotic and eukaryotic microorganisms, corresponding to numerous different taxa, inhabiting a single gram of soil [8]. Fungi are the dominant eukaryotic lineage in terms of biomass in soil, where they play key roles as decomposers, pathogens, and mycorrhizal mutualists [9]. However, with less than 100,000 described species out of the 1.5 million estimated species [10], [11], the fungal kingdom represents one of the least well investigated parts of the world’s biodiversity. The claim that the “dream of many microbiologists is to take a DNA sample from the soil, and understand what species are there, and which are beneficial” [12] seems, therefore, particularly challenging for mycologists, because of fungal inconspicuous nature, their habitats inaccessibility and the difficulty to study the unculturable fungi [13]. In many soils, the biomass of fungi exceeds that of all other soil organisms combined (excluding plant roots) by an order of magnitude. In soil the primary and best known role of fungi is the decomposition and mineralization of complex, recalcitrant compounds of plant and animal origins, such as cellulose, hemicellulose, lignin, and chitin [14], [15]. Other major roles of soil fungi include their involvement in beneficial and detrimental symbioses with plant roots. These range from the mutually beneficial mycorrhizae that enable land plants to exist in the face of nutrient and water limitations and other stresses to the plant pathogens [16]. Moreover, fungi play a key role in controlling the structure and soil water content and in regulating the aboveground biodiversity [17]. Therefore, there is a growing interest in assessing soil biodiversity for the preservation and improvement of ecosystem services as biodiversity is closely linked with soil biological functioning [18]. Due to their large number of species, specialization, and important ecological functions, fungi are also excellent bioindicators. The value of fungi as bio-indicators of environmental quality was demonstrated for wood-inhabiting fungi for undisturbed forest sites [19], [20]; for ectomycorrhizal fungi for air pollution stress in forests [21]–[24], and for saprotrophic fungi for the duration of undisturbed grassland use [25], [26]. In addition, the damage to the fungal contingent, due to atmospheric pollution, has been reported to precede the decline of forest community of about 10 years [22].

For these reasons, investigating the fungal diversity and the ecological factors that underlie the dynamics of fungal communities becomes crucial for the ecological characterization of any given site. In this context, high-throughput amplicon pyrosequencing targeting the ITS region of the fungal rDNA represents a powerful approach to the analysis of soil fungal biodiversity [27] and a good way to detect key or relevant fungal taxa whose presence reflects changes at above-ground level.

Against this background, the present study examines fungal biodiversity in a set of Italian soils subjected to different anthropogenic impact in a typical Mediterranean landscape dominated by *Quercus suber* L. The agro-silvo-pastoral system is similar to those called “*dehesa*” (Spain) or “*montados*” (Portugal) of the southwest of the Iberian Peninsula. Similar conditions are also present in Mulga Lands in eastern Australia as well as in eastern savannas of the United States. The examined areas range from a cork-oak forest under minimum disturbance to agricultural plots with monoculture crops, passing through areas with managed vineyards and pastures. These traditional agro-silvo-pastoral systems are located in Sardinia (Gallura, Berchidda, Italy) and represent a sustainable balance between human activities and natural resources that have created a landscape of high heterogeneity and cultural value, whose importance has been recognized at the European level [28], [29]. However, concerns about the future of such ecosystems are growing as they progress along the trajectory to biological impoverishment, and research into their biodiversity and conservation are urgent issues. Therefore, the aims of this study are to provide an overview of soil fungal diversity at the examined sites and to detect changes in community compositions following different land-uses. The results obtained provide backbone data on the impact of environmental conditions (soil types, cover vegetation, and human activities) on the distribution of the fungal genetic potential (biodiversity) in soil communities for this Mediterranean region.

## Materials and Methods

### Study Site

The study area is located in the northern hills of Sardinia (Gallura), Italy. The Berchidda site (Olbia-Tempio, 40°30′13.37′′N, 9°47′00.56′′E) is made up of Palaeozoic intrusive rocks (inequigranular monzogranites). The altitude ranges from 275 m to 300 m. The dominant soil reference group is Leptic Cambisols, with loamy-sand texture and pH values ranging from 5.0 to 6.5 (Table 1) [30]. This area is referred to as a meso-mediterranean, subhumid phytoclimatic belt with annual rainfall averages of 862 mm (5% summer rainfall percentage), and the mean temperature is 13.8°C [31].

**Table 1:** Main features of the five investigated Sardinian soils.

	Sand (g/kg)	Silt (g/kg)	Clay (g/kg)	Field capacity (% vol)	Soil pH	Org C (g/kg)	tot N (g/kg)	CEC (meq/100g)
**CV**	484	84	114	14.6	6.2	12.3	1.01	14.92
**TV**	543	48	122	14.6	5.1	14.3	0.99	12.45
**MM**	411	138	128	18.3	5.6	19	1.25	15.01
**PA**	395	132	136	20.3	5.4	23.7	2.22	19.25
**CO**	450	134	130	25.7	5.9	59.24	5.39	28.2

Modified from Pastorelli et al. [30]. TV, tilled vineyard; CV, covered vineyard; MM, managed meadow; PA, pasture; CO, cork-oak formation. CEC, Cation-Exchange Capacity.

In the past, the area was covered by cork-oak forests which gradually were subjected to increasing understorey grazing and usage for the extraction of cork. Today, there are five dominant soil use types: 1) tilled vineyard (TV), 2) non-tilled cover cropped vineyard (CV) with the following cover crop species: *Geranium molle*, L., *Hordeum leporinum Link*, *Medicago arabica* (L.) Huds, *Medicago polymorpha* L., *Trifolium nigrescens* Viv., and *Avena barbata* Potter; 3) managed meadow (MM) with the following main forage crops: *Avena sativa* L., *Lolium multiflorum* Lam., and *Trifolium michelianum* Savi; 4) pasture or grazed secondary grassland (PA) dominated by pasture or grass species such as *Avena sp.*, *Vulpia sp.*, *Trifolium subterraneum* L., and *T. nigrescens* Viv., with a low cork-oak tree density; and 5) cork-oak formation (CO) dominated by shrub cover and scattered cork-oak trees. Grazing systems (particularly dairy sheep, meat cattle, and goats) are widely present, occurring in all areas except CV. The cork-oak formation, pasture, and managed meadow have been converted to the current use and maintained unchanged for more than 30 years, whereas the non-tilled cover cropped vineyard and the tilled one were planted in 1985 and 1994, respectively. The five soils are located inside an area of 1×1.5 km^2^.

All necessary permits were obtained for the sampling in the considered study site. The soil sampling was made in the frame of a national research project (SOILSINK Project). The responsible of study site was Prof PP Roggero.

### Soil Sampling, DNA Extraction, PCR, and Preparation of the Amplicon Libraries

In May 2007, five soil core samples (5 cm diameter at 20 cm depth) were taken from each of the five locations, using a 5-on-dice sampling pattern with ca. 70 m distance between each sampling point. Sampling was performed at 20 cm depth, where most fungal activity is known to occur [32]. The 25 soil samples were independently packed in ice upon collection and transported to the laboratory for DNA extraction. The soil samples were sieved (2 mm) to remove fine roots and large organic debris and stored at −80°C. Twenty-five soil DNA extractions were performed from 0.5 g of mixed soil with the FastDNA Kit (MP Biomedicals, LLC, OH, USA). The quality and quantity of DNA samples was assessed through gel electrophoresis of 5 µl subsamples on 1.5% agarose gel and analysis with the ND-1000 Spectrophotometer NanoDrop® (Thermo Scientific, Wilmington, Germany).

Two sets of primers, ITS1F-ITS2 [33] and ITS3-ITS4 [34], were used to amplify the two spacers ITS1 and ITS2 of the internal transcribed spacer (ITS) rDNA region. A total of 50 independent PCR amplifications were performed: 25 with the ITS1F-ITS2 and 25 with the ITS3-ITS4 primer set. The DNA amplifications were performed from an equivalent amount of DNA to that found in 2.5 grams of each soil type. To make sure that the communities were sampled deeply enough, we pooled the PCR products independently amplified from the 5 soil samples as obtained from the same location (TV, CV, MM, PA, and CO).

The first primer set is fungus-specific for the ITS1 region and amplifies a fragment of *c.* 400 bp: ITS1F (5′-(*A*)CTTGGTCATTTAGAGGAAGTAA-3′) and ITS2 (5′-(*B*)GCTGCGTTCTTCATCGATGC-3′). The second set is eukaryote-specific for the ITS2 region and amplifies a fragment of *c.* 350 bp: ITS3 (5′-(*A*)GCATCGATGAAGAACGCAGC-3′) and ITS4 (5′-(*B*)TCCTCCGCTTATTGATATGC-3′). The *A* and *B* sequences fused to the 5′ primer ends represent the pyrosequencing adapters (19 bp): GCCTCCCTCGCGCCATCAG and GCCTTGCCAGCCCGCTCAG, respectively.

The polymerase chain reactions (PCRs) contained 17.1 µl of sterile water, 2.5 µl 10X of reaction buffer (Roche), 2.5 µl of each deoxyribonucleotide triphosphate (dNTP 2.0 µM), 0.5 µl of each primer (10 µM), 0.4 µl of DNA polymerase (High Fidelity Taq, Roche), and 2 µl of DNA template in a final volume of 25 µl.

The DNA was amplified using a T3000 thermal cycler (Biometra, Göttingen, Germany) using the program: initial denaturation at 94°C for 3 min, followed by 35 cycles of denaturation at 94°C for 45 s, annealing at 60°C for 45 s, extension at 72°C for 1 min, and a final extension at 72°C for 7 min with a ramp of 3°C/s.

Twenty-five independent amplifications (five for each soil type) were conducted for the ITS1F/ITS2 and ITS3/ITS4 sets. The PCR products obtained with the two primer pairs were purified with the Agencourt® AMPure® Kit (Beckman Coulter, CA, USA) and pooled to generate ten libraries (five for each of ITS1F/ITS2 and ITS3/ITS4). The quality of the samples was assessed through: (a) gel electrophoresis of 5 µl subsamples on 1.5% agarose gel, (b) evaluation of the AD260/280 ratio calculated using the ND-1000 Spectrophotometer NanoDrop® (Thermo Scientific, Wilmington, Germany); and (c) analysis with the Experion™ System (Bio-Rad, Hercules, CA, USA), using a DNA1K Chip.

In order to create equimolar mixtures of multiple amplicons (amplicon libraries) for 454 pyrosequencing, the ten pooled samples were quantified by the ND-1000 Spectrophotometer NanoDrop®. Five final amplicon libraries (TV, CV, MM, PA, and CO), containing ∼10^10^ molecules/µl of each primer set amplification, were generated. The samples were stored at −20°C and sent to BMR Genomics s.r.l. (Padua, Italy) for pyrosequencing by means of a Genome Sequencer FLX System platform (454 Life Science Branford, CT, USA). The samples were processed together with other soils and they occupied five lanes out of the sixteen available in the GS-FLX System. Equimolar concentrations of four DNA fragments from each of the five soils were combined and loaded in one lane; in addition to the ITS1 and ITS2 fragments considered in this work, each lane was loaded with fragments amplified with two primer sets specific for arbuscular mycorrhizal fungi, whose diversity was described in a previous paper [35].

### Bioinformatic Processing and Statistical Analysis

The ITS1 and ITS2 fragments were located and extracted using Nilsson *et al*. [36] to reduce the noise imparted by the neighbouring, very conserved genes and to filter out sequences that were not from the ITS region [37]. Sequences shorter than 100 bp, as well as sequences with more than one DNA ambiguity symbol [38], were dismissed. The average lengths of the sequences after removal of the conserved neighbouring genes and the removal of overly short sequences were 162 bp (ITS1) and 117 bp (ITS2). The sequences were then subjected to 97% similarity single-linkage clustering using TGICL as implemented in the ITS pyrosequencing pipeline of Tedersoo et al. [39] (default settings) using GenBank [40] and UNITE [41] as taxonomic reference databases. UNITE covers ectomycorrhizal fungi well; the non-ECM core sequences in UNITE currently correspond to some 5% of the total number of sequences, a small number but large enough to make it a relevant resource for comparison.

Representative sequences of each fungal OTU (Operational Taxonomic Unit) [42] were submitted to EMBL Nucleotide Sequence Database (accession numbers FR838022-FR838930).

The rarefaction analysis was performed using ANALYTIC RAREFACTION v.1.3 (Hunt Mountain Software, Department of Geology, University of Georgia, Athens, GA, USA).

The matrices of ITS1 and ITS2 OTU relative abundances (calculated for each OTU on the total sequences of each soil dataset) were used to perform the following multivariate analyses: a) classification analysis (UPGMA, chord distance as the resemblance measure, 100 bootstrap replicates) [43], b) ordination analysis by means of Principal Coordinate Analysis (PCoA: symmetric scaling with species score divided by standard deviation, square-root transformation, centering samples by samples, and centering species by species) [44]. Classification analyses were performed using SYN-TAX 2000 - Hierarchical Classification, while ordinations were performed using CANOCO 4.5. Low-abundance OTUs (OTUs with relative abundances <1% in all soil samples, including singletons) were excluded from the analyses as have been done in other studies [39], [45].

Differences in the proportions of OTUs and sequences of fungal functional and ecological groups, as well as taxonomic groups highly represented in at least one soil, were tested for significance, in pairwise comparisons (degrees of freedom  =  1) between the different soils, by means of the Chi-square test (performed using StatView SE+ v1.02).

## Results

### Pyrosequencing, Clustering, and BLAST Results

**Figure 1:**
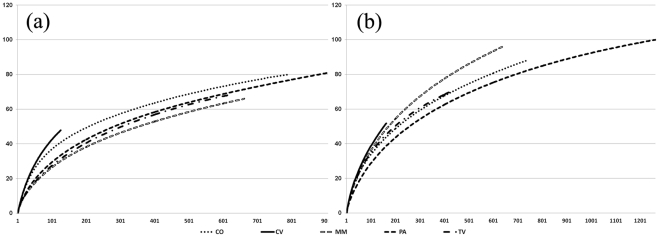
Rarefaction curves describing observed fungal richness. Rarefaction curves indicating the observed number of operational taxonomic units (OTUs) at a genetic distance of 3% related to the number of sequences retrieved in each of the five different soils with ITS1 (**a**) and ITS2 (**b**) primer set, respectively. TV, tilled vineyard; CV, covered vineyard; MM, managed meadow; PA, pasture; CO, cork-oak formation. X-axis  =  sequences number; Y-axis  =  OTUs number.

A total of 8817 sequences were obtained from the pyrosequencing run: 4740 ITS1 (average length 162 bp) and 4077 ITS2 sequences (average length 117 bp). All further analyses were based on fragments containing only the ITS1 and ITS2, respectively; all parts (>18 bp) of nSSU, 5.8S, and nLSU were removed or they would have interfered with the clustering and BLAST searches [36]. Sequences shorter than 100 bp after the extraction were discarded: 4317 ITS1 and 3961 ITS2 sequences remained for further analyses. Among these, 3097 and 3234 were identified as fungal sequences and were of sufficient quality for further analyses. Sequences were assigned to OTUs based on 97% sequence similarity: 386 and 506 unique fungal OTUs were recovered from the two sequence datasets. Of these, 230 (59.6%) and 336 (66.4%) were singletons. With both primer sets, the highest number of fungal sequences and OTUs were retrieved from the pasture soil, while the lowest numbers were obtained in the covered vineyard (Table 2). The ratio of Ascomycota/Basidiomycota was calculated with both OTU sets and a similar trend was observed: the lowest values in cork-oak formation and pasture soils and the highest values in managed meadow and tilled vineyard soils (Table 2). Rarefaction analysis of the fungal OTUs assigned at 97% similarity indicated undersampling of actual richness (Figure 1).

**Table 2:** Taxonomic distribution of sequences and OTUs retrieved with the ITS1F-ITS2 (ITS1 region) and ITS3-ITS4 (ITS2 region) primer set in the five Sardinian soils.

		ITS1F-ITS2 (ITS1 region)									
		CV		TV		MM		PA		CO	
		Sequences (no.)	OTUs (no.)	Sequences (no.)	OTUs (no.)	Sequences (no.)	OTUs (no.)	Sequences (no.)	OTUs (no.)	Sequences (no.)	OTUs (no.)
Ascomycota		84	36	255	58	324	55	621	52	262	42
Basidiomycota		25	9	116	9	224	12	211	29	462	35
Chytridiomycota		0	0	4	2	0	0	0	0	0	0
Glomeromycota		1	1	0	0	0	0	2	1	0	0
Zygomycota		16	5	238	8	114	5	73	3	65	6
Asco/BasidiomycotaOTU ratio		4.0		6.4		4.6		1.8		1.2	
	**ITS3-ITS4 (ITS2 region)**										
	**Sequences (no.)**		**OTUs (no.)**	**Sequences (no.)**	**OTUs (no.)**	**Sequences (no.)**	**OTUs (no.)**	**Sequences (no.)**	**OTUs (no.)**	**Sequences (no.)**	**OTUs (no.)**
Ascomycota	78		38	283	56	548	92	646	80	326	59
Basidiomycota	29		8	55	11	39	15	574	36	381	34
Chytridiomycota	0		0	0	0	0	0	0	0	0	0
Glomeromycota	4		3	0	0	0	0	0	0	0	0
Zygomycota	52		9	100	10	50	6	42	3	27	11
Asco/BasidiomycotaOTU ratio	4.8			5.1		6.1		2.2		1.7	

Asco/Basidiomycota ratios are reported for each of the fungal communities retrieved with both primer sets in all the soils. TV, tilled vineyard; CV, covered vineyard; MM, managed meadow; PA, pasture; CO, cork-oak formation.

**Figure 2:**
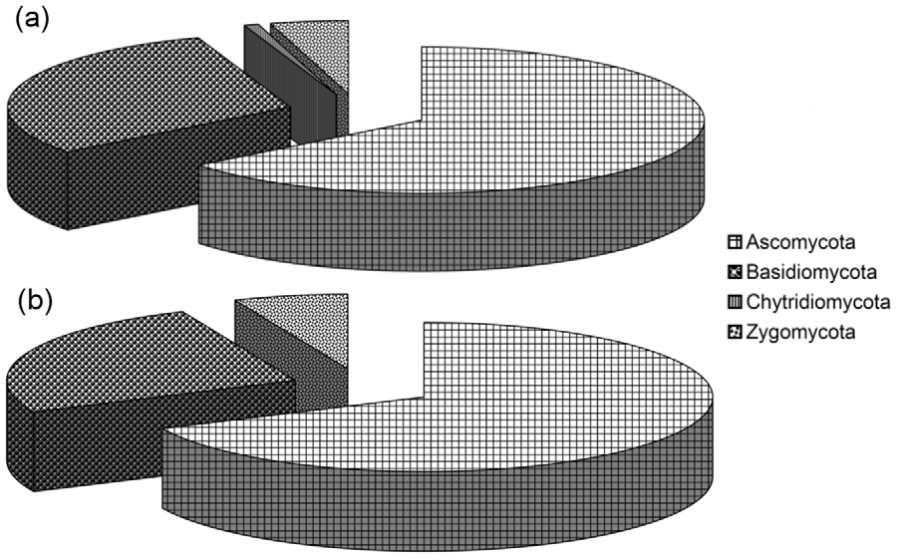
Taxonomical distribution of retrieved fungi. Proportion of the (**a**) 240 ITS1 and (**b**) 326 ITS2 fungal operational taxonomic units (OTUs) assigned to the different fungal phyla. Ascomycota were dominant (64.5% of total ITS1 OTUs and 68.0% of total ITS2 OTUs), followed by Basidiomycota (30.3% and 25.4%), Zygomycota (4.5% and 6.5%), and Chytridiomycota (0.6% and 0%).

BLAST searches [46] revealed that sequences and OTUs belonging to the phylum Ascomycota were dominant, followed by Basidiomycota, Zygomycota and Chytridiomycota (Figure 2). Eighty-five genera were recovered by both sets of primers (Tables S1, S2). Among them, the genera with the highest OTU number were *Penicillium* (14 and 16 OTUs with ITS1 and ITS2 primers, respectively) and *Cryptococcus* (10 OTUs with both primer pairs). By contrast, 51 other genera (e.g., *Lactarius* and *Phialophora*) were exclusively found with the ITS1 primers, while 71 other genera (e.g., *Geoglossum* and *Torula*) were exclusively found with the ITS2 primer set.

### Multivariate Analyses

**Figure 3:**
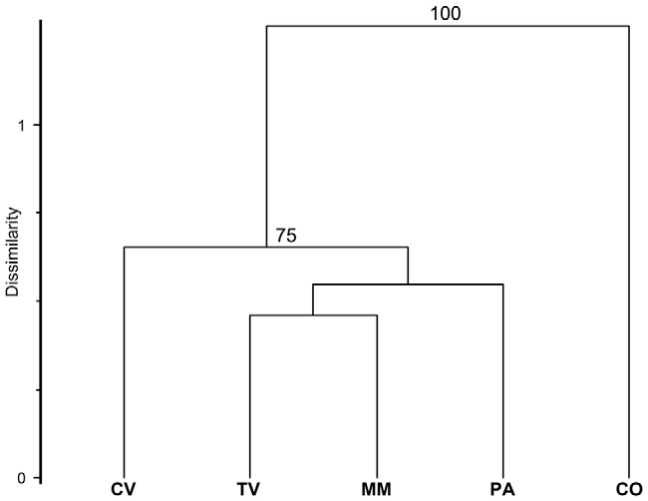
Dendrogram showing distribution of fungal assemblages. Classification analysis (UPGMA, chord distance as the resemblance measure) on the basis of OTU relative abundances data. Numbers on branches represent the bootstrap confidence percentage (100 replicates). TV, tilled vineyard; CV, covered vineyard; MM, managed meadow; PA, pasture; CO, cork-oak formation.

Classification analysis (chord distance) of ITS1 OTU relative abundances separated the forest soil [cork oak formation (CO)] from non-forest soils (100% bootstrap support) (Figure 3). The same result was obtained with the ITS2 OTUs (data not shown).

**Figure 4:**
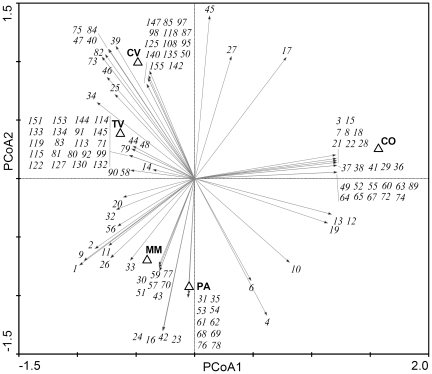
Principal Coordinate Analysis (PCoA) of most abundant fungal OTUs. Ordination on axes 1 and 2 of soils on the basis of the ITS1 OTU presence (PCoA). High-abundance OTUs (>1% in at least one soil) are considered for the statistical analysis (list of these OTUs is reported in table S1). Low-abundance OTUs (including singletons) are excluded. TV, tilled vineyard; CV, covered vineyard; MM, managed meadow; PA, pasture; CO, cork-oak formation.

Principal Coordinates Analysis (PCoA) was carried out to ordinate ITS1 OTUs obtained from the five Sardinian soils (Figure 4) based on OTU relative abundances. On the first axis (accounting for 42.7% of the total variance) the cork-oak soil (CO) was clearly separated from the other four non-forest soils (CV, TV, MM, and PA) (Figure 4). This separation was determined by several OTUs [for example: *Cortinarius stillatitius* (OTU 3), *Hygrophorus persoonii* (OTU 7), *Microglossum olivaceum* (OTU 8), *Russula virescens* (OTU 22) (Figure 4; Table S1)] which featured the highest positive correlation with axis 1, being exclusive of/more represented in the cork-oak soil, as well as other OTUs, such as *Fusarium oxysporum* (OTU 1), *Cryptococcus phenolicus* (OTU 2), and *Cladosporium cladosporioides* (OTU 9), showed the highest negative correlation with the same axis, being absent or less represented in CO than in the other four soils.

The second axis (accounting for 25.8% of the total variance) mainly separated grass soils (PA and MM) from vineyard soils (CV and TV). *Neonectria radicicola* (OTU 45) featured the highest positive correlation with axis 2, being more abundant in vineyard than in grass soils (Table S1). The highest negative correlation with axis 2 was instead observed for the following four OTUs, which were exclusively found in grass soils: *Cryptococcus victoriae* (OTU 16), *Thelebolus microsporus* (OTU 23), *Phoma americana* (OTU 24), and *Talaromyces flavus* (OTU 42) (Figure 4; Table S1). Some OTU vectors were located around the centre of the diagram (Figure 4), being detected in all sites (e.g., OTU 1, 2, and 40). Some of them are known as largely widespread species: for example, *Fusarium oxysporum* (OTU 1) and *Chaetomium globosum* (OTU 40). Similar PCoA results were obtained for the ITS2 fungal OTUs (data not shown).

### Fungal Communities in the Five Sardinian Land-use Backgrounds

A core set of fungal OTUs was detected in all the five Sardinian soils: 4 ITS1 (*Fusarium oxysporum*, *Cryptococcus phenolicus*, *Chaetomium globosum*, *Neonectria radicicola*) and 9 ITS2 (*Chaetomium globosum*, *Fusarium oxysporum*, *Torula herbarum*, *Mortierella elongata*, *Cladosporium cladosporioides*, *Cryptococcus elinovii*, *Thielavia terricola*, *Penicillium restrictum*, *Cenococcum geophilum*) OTUs, respectively (Tables S1 and S2). Many of these OTUs were rich in term of sequence numbers; *Fusarium oxysporum*, e.g., included 642 ITS1 and 434 ITS2 sequences (26.3% and 14.0% of total ITS1 and ITS2 sequences, respectively). However, proportions of sequences assigned to these OTUs could vary significantly among the different environments (see below).

#### Cork oak formation (CO)

The fungal sequence datasets obtained from the cork oak forest soil were unique in being dominated by OTUs, assigned to *Cortinarius* and *Hygrophorus* species (accounting for 26.8% and 6.5% of ITS1, and 14.2% and 16.8% of ITS2 total sequences, respectively), which were exclusively obtained from this soil. Significantly higher numbers of both ITS1 and ITS2 sequences of Basidiomycota (P<0.001, Chi-square test) and, correspondingly, lower numbers of both ITS1 and ITS2 sequences of Ascomycota (P<0.001, Chi-square test) were indeed obtained from CO than the other four soils (Table 2). Significant lower proportions of ITS1 and ITS2 sequences of *Fusarium oxysporum*, a taxon shared by all soils, were obtained from CO than the other environments (P<0.001, Chi-square test. Basidiomycota sequences accounted for 61.7% and 40.8% of total ITS1 and ITS2 sequences from CO, respectively. These were mostly strictly ectomycorrhizal taxa (91.% and 96.2% of ITS1 and ITS2 Basidiomycota sequences, respectively), including *Cortinarius*, *Hygrophorus*, *Russula*, *Inocybe*, *Gymnomyces*, *Amanita* and *Sebacina* spp. (obtained with both ITS1 and ITS2 amplification), as well as *Clavulina*, *Gyroporus*, *Hymenogaster*, *Lactarius* and *Thelephora* spp., which were instead only obtained with the ITS1 marker.

Another peculiar feature of the fungal sequence datasets from the CO soil was the exclusive occurrence of *Geoglossaceae*, which are typical colonizers of low-input grazing areas (*P*<0.001, Chi-square test; 6.2% and 7.7% total ITS1 and ITS2 sequences, respectively).

Overall, the CO datasets featured 38 ITS1 and 29 ITS2 non-singleton exclusive OTUs (encompassing 65.1% and 55.3% of total ITS1 and ITS2 sequences, respectively).

#### Pasture (PA)

The ITS1 and ITS2 sequence datasets from pasture were dominated by sequences of taxa shared by all soils (*Fusarium oxysporum*, which accounted for 43.7% ITS1 total sequences, and C*haetomium globosum*, that accounted for 35.0% of ITS2 total sequences). However, a significant higher proportion of ITS1 sequences of *Fusarium oxysporum* was obtained from PA than the other soils (P<0.001, Chi-square test). As the CO datasets, the PA datasets featured a higher number of sequences of strictly ectomycorrhizal Basidiomycota than the datasets from the managed meadow and the two vineyards (P<0.05, Chi-square test; 24.7% and 8.3% of total ITS1 and ITS2 sequences from PA, respectively; 59.1% and 50.0% of ITS1 and ITS2 Basidiomycota sequences, respectively). However, fewer OTU and sequence numbers of ectomycorrhizal taxa were obtained from PA than CO (P<0.05, Chi-square test). Furthermore, with the exceptions of *Russula aeruginea*, *R. odorata*, *Cortinarius casimiri*, *C. gallurae* and *C. rubricosus* (that were also retrieved from the oak forest soil), and *Geastrum coronatum* (which was also retrieved from the managed meadow soil), the PA ectomycorrhizal taxa (*Boletus*, *Cortinarius*, *Clavulina*, *Entoloma*, *Geastrum*, *Gymnomyces*, *Laccaria*, *Russula*, *Scleroderma* and *Tomentella* spp., which accounted for 14.6% and 4.2% of ITS1 and ITS2 total sequences, respectively) were exclusively obtained from the latter soil. A higher number of ITS2 sequences assigned to coprophilous fungi (mostly to *Thelebolus microsporus*) was obtained from PA than CO (P<0.001, Chi-square test).

Fourteen ITS1 and 25 ITS2 non-singleton exclusive OTUs (accounting for 7.7% and 7.1% of total ITS1 and ITS2 sequences, respectively) were obtained from PA.

#### Managed meadow (MM)

*Fusarium oxysporum* was the dominant taxon in both ITS1 and ITS2 MM datasets (32.4% and 20.8% ITS1 total sequences, respectively). Several taxa [4 ITS1 OTUs (that accounted for 3.2% and 3.3% total ITS1 sequences, and included the coprophilous fungus *Thelebolus microsporus*) and 10 ITS2 OTUs (2.8% and 2.0% total ITS2 sequences)] were exclusively found in the MM and PA soils. The ITS1 MM dataset was distinguished from the PA dataset by the lower proportions of Ascomycota sequences and Basidiomycota OTUs and, vice versa, the higher proportions of Basidiomycota sequences and Ascomycota OTUs (P<0.05, Chi-square test), while the ITS2 dataset was distinguished by the lower number of Basidiomycota OTUs (P<0.05, Chi-square test). Furthermore, the MM datasets featured 9 ITS1 and 9 ITS2 non-singleton exclusive taxa (accounting for 6.7% and 6.5% of total ITS1 and ITS2 sequences, respectively).

#### Covered vineyard (CV) and tilled vineyard (TV)

The ITS1 OTU comprising the highest number of sequences was *Fusarium oxysporum* for both vineyards (24.3% and 25.7% ITS1 total sequences, respectively), whereas the ITS2 dataset was dominated by *Mortierella elongata* for the covered vineyard (18.1% total sequences) and by *Chaetomium globosum* (14.6%) for the tilled vineyard. However, a higher proportion of *Fusarium oxysporum* ITS2 sequences was obtained from the TV than the CV soil (9.3% vs 2.8% total sequences, respectively). The datasets from the two vineyards shared a higher proportion of both ITS1 and ITS2 sequences assigned to Zygomycota (accounting for 12.6% and 7.0% of total ITS1 sequences in CV and TV, respectively, and 30.7% and 21.9% of total ITS2 sequences in CV and TV, respectively) than in the other soils (where Zygomycota accounted for 0.9–4.3% of total ITS1 sequences, and 3.0–7.8% of total ITS2 sequences, respectively; P<0.001, Chi-square test). However, a higher proportion of ITS1 and ITS2 Zygomycota sequences was obtained from CV than TV (P<0.05, Chi-square test). Five ITS1 taxa (accounting for 8.7% and 12.5% total CV and TV sequences, respectively) and 3 ITS2 taxa (3.5% and 3.1% total CV and TV sequences, respectively) were exclusively found in the CV and TV soils. Five ITS1 (9.7% total sequences) and 9 ITS2 (17.4% total sequences) taxa were instead exclusively obtained from the CV soil, while 20 ITS1 (20.3% total sequences) and 13 ITS2 (8.8% total sequences) taxa were instead exclusively obtained from the TV soil. Coprophilous fungi (*Podospora* spp.) were exclusively found in the TV ITS1 dataset (5.7% total sequences).

## Discussion

Fungi are one of the largest and most diverse kingdoms of eukaryotes and are important biological components of all terrestrial ecosystems. Understanding how symbiotic, saprotrophic and pathogenic fungi achieve their lifestyle is crucial for understanding their ecological functions and their impact on plant communities [13].

It is difficult to overemphasize the importance of fungi in soil due to their inconspicuous nature, their habitats inaccessibility and our inability to cultivate many of them. However, Next Generation Sequencing DNA surveys bypassed some of these limitations and allowed us to determine the biodiversity and the dynamics of many different fungal soil communities [39], [47]–[50].

In this study we combined a more traditional taxonomical approach with a detailed evaluation on the ecological traits of the retrieved fungal OTUs. Data analyses consistently indicated a sharp distinction of the fungal community of the cork oak forest soil from those described in the other soils. This finding confirmed and extended the results obtained on bacterial communities [30] and on arbuscular mycorrhizal fungi [35] in the same soils using pyrosequencing approach.

Pyrosequencing is a powerful alternative to traditional approaches based on cloning and Sanger sequencing in terms of cost, time, and number of sequences obtained [39]. By identifying more taxa, this strategy generates a larger sample of the fungal community at hand than it is normally obtainable using traditional Sanger sequencing. Because of the possible technical and methodological shortcomings, however, it is less well suited for quantitative analysis. For instance, possible primer biases argue against comparison of sequence numbers across the different OTUs. Furthermore, another risk aspect which should be considered is the different numbers of sequences obtained in each of the amplicon libraries. Considering uneven sampling depth, relative abundances (calculated for each OTU on the total sequences of each soil dataset) were taken into account in this paper.

We propose a strategy for assessment of fungal biodiversity based on the use of two target regions (ITS1 and ITS2). The choice of primers is important because it is known that various commonly utilized ITS primers might introduce biases during the amplification of different parts of the ITS region. In this study, the pair of primers ITS1F/ITS2 used for the amplification of the ITS1 region produced less non-fungal sequences in addition to a higher number of sequences than the pair ITS3/ITS4 amplifying the ITS2 region. Given that ITS1F is a fungal specific primer, it is not surprising to find more fungal sequences in the ITS1 region data. As a consequence, the pair of primers ITS1F/ITS2 was more selective. As already reported in Margulies et al. [51] the long fragments inhibit the emulsion-PCR step at the expense of the short PCR amplicons. Thus, the longer fragment of the ITS2 probably inhibited the emulsion-PCR step, at the expense the shorter ITS1 fragment and this could explain the lower number of ITS2 obtained in our and other studies [52]. Moreover, *in silico* PCR analyses performed by Bellemain et al. [53] indicated that ITS3 and ITS4 primers preferentially amplified Ascomycetes, whereas ITS1F preferentially amplified Basidiomycetes. According with these authors and Mello et al. [52], using the ITS2 region we have found a slightly higher number of the Ascomycetes compared to Basidiomycetes.

The effectiveness of this approach is confirmed by the relevant number of fungal taxa exclusively retrieved with each one of the considered target regions. The analysis of different parts of the ITS region in parallel is indeed strongly recommended to get a more reliable assessment of fungal biodiversity [53]. However, some fungal taxa, in particular members of Glomeromycota and Chytridiomycota, were sporadically retrieved with either ITS primer sets, as already highlighted by other studies [32], [48]. In addition, well-populated reference databases such as UNITE [41] are essential for reliable identification of the sequences. Even though UNITE database is curated, its core sequences are mainly ectomycorrhizal, with 5% of sequences belonging to other fungi. The importance of using curated databases for fungal identification is confirmed by the new *Fungal LSU Classifier* tool available in the frame of the Ribosomal Database Project (RDP). The low number of deposited and certified fungal sequences in the public sequence databases represents a limitation for the taxonomical assignment in biodiversity study, especially considering that new high-throughput technologies allow retrieving a large amount of unknown, and probably unculturable, fungi. Another important limitation is the relative short-fragment length obtainable with the GS-FLX Standard Series Kit (250 bp average length, becoming 400 bp with the new Titanium Series Kit) of the 454 pyrosequencing platform. The obtained reads may not always be long enough for accurate species identification, as discussed in other studies [49], [54]. Thus, considerations of putative lifestyle (i.e. coprophilic activity, symbiotic ability, etc.) of the investigated organisms, in addition to the ‘‘taxonomic approach’’, may prove useful in the assessment and monitoring of biodiversity. A multi-disciplinary exchange involving taxonomists, molecular biologists, ecologists, and bioinformaticians will be necessary to overcome this lack [55], [56].

Mostly likely, such results reflect the impact of both land-use and vegetation type and coverage on soil microbial communities, the oak forest being a more natural environment than the other, more anthropogenic and crop-covered areas [30], [35], [57]. At the same time, each of the five soils harboured fungi that could mirror the ecological traits of the environment where they were retrieved. Hence, the high proportion of ectomycorrhizal Basidiomycota OTUs in the cork-oak formation is not surprising, since this habitat is characterized by tree coverage, mainly *Quercus suber* L. which typically establish this kind of symbiosis. Similarly, the pasture soil, where sporadic oak trees occurred, featured a high proportion of ectomycorrhizal fungal sequences. Furthermore, the forest soil, which is subjected to cyclic disturbances (winter cattle grazing), featured several taxa, such as *Geoglossaceae* and *Hygrophoraceae*, able to adapt their dynamics with this disturbance. Therefore, they are considered as indicators of natural grasslands [25] and their presence/absence is often used as an indicator of the extent of restoration or degradation of habitat value [58]. Other features of the fungal assemblages retrieved can be easily related to the above-ground settings. For instance, the abundance of coprophilic fungi in pasture and managed meadow soils can be traced back to the high grazing sheep pressure throughout the year. Similarly, the occurrence of coprophilous fungal sequences in the tilled vineyard mirrors the sheep winter grazing in such environment. Large numbers of yeasts and yeast-like fungi, known to be associated with grapevine fields and wine production [59], [60], were obtained from the vineyard soil.

Beyond the occurrence of typical fungal OTUs in each environment, it is also important to underline the presence of ubiquitous fungi shared by all soils. These belonged to a low number of OTUs, but they are the most abundant in terms of sequence number. This observation suggests that each environment has a core set of generalist fungi able to colonize the soil regardless of its ecological properties.

In conclusion the combined pyrosequencing-based analysis of both internal transcribed spacer regions provided insights on the soil fungal communities associated with different land-uses. Therefore, investigating the below-ground microbial community may provide useful elements on the above-ground features such as vegetation coverage, and agronomic procedures, allowing to assess the cost of anthropogenic land use to hidden diversity in soil. Lastly, the provided datasets may contribute to future searches for fungal bio-indicators to use as biodiversity markers of a specific site and of a land-use.

## Supporting Information

Table S1 **ITS1 OTU abundance in terms of sequence number in the five Sardinian soils.**
(PDF)

Table S2 **ITS2 OTU abundance in terms of sequence number in the five Sardinian soils.**
(PDF)
