# Supplementary material for: Unravelling Soil Fungal Communities from Different Mediterranean Land-Use Backgrounds
Source: PLoS One. 2012 Apr 20;7(4):e34847. doi: 10.1371/journal.pone.0034847 (PMC3335027; doi:10.1371/journal.pone.0034847)
Supplement: Table S1 — ITS1 OTU abundance in terms of sequence number in the five Sardinian soils. (PDF) [file pone.0034847.s001.pdf]

Table S1

	Phylum	Putative lifestyle[1]	OTU number	Closest accession number	Habit	CV (no. seq.)	TV	MM	PA	CO
Podospora glutinans	Ascomycota	coprophile[2]	48*	AY615208	filamentous	0	8	1	0	0
Podospora intestinacea	Ascomycota	coprophile	80*	AY515363	filamentous	0	4	0	1	0
Podospora intestinacea	Ascomycota	coprophile	83*	AY515363	filamentous	0	4	0	0	0
Podospora glutinans	Ascomycota	coprophile	92*	AY615208	filamentous	0	2	2	0	0
Sordaria tomentoalba	Ascomycota	coprophile	129	AY681195	filamentous	0	0	2	0	0
Podospora vesticola	Ascomycota	coprophile	154	AY515365	filamentous	0	0	2	0	0
Thelebolus microsporus	Ascomycota	coprophile	23*	DQ028268	filamentous	0	0	8	16	0
Hydnobolites cerebriformis	Ascomycota	ectomycorrhizic[1]	106	EU784272	filamentous	0	0	0	0	3
Tuber indicum	Ascomycota	ectomycorrhizic	115*	AF132502	filamentous	0	3	0	0	0
Tuber indicum	Ascomycota	ectomycorrhizic	140*	U89362	filamentous	2	0	0	0	0
Cordyceps chlamydosporia	Ascomycota	entomogenous	58*	AB378543	filamentous	1	0	4	1	1
Metarhizium anisopliae	Ascomycota	entomopathogen	47*	FJ609306	filamentous	3	7	0	0	0
Oidiodendron pilicola	Ascomycota	ericoid endophye/ectomycorrhizic	136	AF062787	filamentous	0	0	0	0	2
Hypocrea lixii	Ascomycota	mycoparasite	17*	AJ507064	filamentous	1	2	0	0	24
Hypocrea lixii	Ascomycota	mycoparasite	100	FJ884157	filamentous	0	0	0	0	3
Bionectria ochroleuca	Ascomycota	mycoparasite	116	GQ495672	filamentous	0	0	1	1	1
Leptodontidium orchidicola	Ascomycota	orchid endophyte	25*	AF214577	filamentous	1	21	0	0	0
Phoma exigua	Ascomycota	phytoparasite	14*	GU062320	filamentous	4	0	0	25	0
Phoma americana	Ascomycota	phytoparasite	24*	FJ426972	filamentous	0	1	9	13	0
Plectosphaerella cucumerina	Ascomycota	phytoparasite	33*	GU062300	filamentous	1	0	11	5	0
Pyrenophora avenae	Ascomycota	phytoparasite	43*	FJ907537	filamentous	0	0	6	0	0
Neonectria radicicola	Ascomycota	phytoparasite	45*	FJ861415	filamentous	2	2	1	1	5
Sclerotinia minor	Ascomycota	phytoparasite	46*	AB516661	filamentous	1	9	0	0	0
Phoma boeremae	Ascomycota	phytoparasite	64*	FJ426982	filamentous	0	0	0	0	7
Neonectria macrodidyma	Ascomycota	phytoparasite	75*	AY677281	filamentous	2	2	1	0	1
Phoma chrysanthemicola	Ascomycota	phytoparasite	91*	FJ426987	filamentous	0	4	0	0	0
Phaeosphaeria oryzae	Ascomycota	phytoparasite	120	AF250833	filamentous	0	0	2	0	0
Acrostalagmus luteoalbus	Ascomycota	phytoparasite	127*	AJ292420	filamentous	0	2	0	0	0
Pochonia bulbillosa	Ascomycota	phytoparasite	139	AB378552	filamentous	0	0	0	2	0
Fusarium oxysporum	Ascomycota	phytoparasite/saprobe	1*	GQ922563	filamentous	25	81	150	359	27
Fusarium solani	Ascomycota	phytoparasite/zoopathogen	95*	GQ922557	filamentous	1	0	2	0	0
Otidea cochleata	Ascomycota	probably ectomycorrhizic	89*	EU784386	filamentous	0	0	0	0	4
Hypocrea pachybasioides	Ascomycota	saprobe	5	GU062213	filamentous	0	0	0	0	2
Microglossum olivaceum[3]	Ascomycota	saprobe	8*	EU784374	filamentous	0	0	0	0	46
Humicola grisea var. *grisea*	Ascomycota	saprobe	11*	AY706334	filamentous	0	17	7	9	2
Penicillium urticae	Ascomycota	saprobe	12*	GQ389620	filamentous	0	0	0	5	30
Penicillium melinii	Ascomycota	saprobe	21*	FJ230993	filamentous	0	0	2	3	20
Chaetomium funicola	Ascomycota	saprobe	31*	GQ996574	filamentous	0	0	0	18	0
Ulocladium tuberculatum	Ascomycota	saprobe	32*	FJ914692	filamentous	0	8	8	1	0
Penicillium skrjabinii	Ascomycota	saprobe	35*	EU427287	filamentous	0	0	1	14	0
Chaetomium globosum	Ascomycota	saprobe	40*	GQ355338	filamentous	2	5	1	4	1
Talaromyces flavus var. *flavus*	Ascomycota	saprobe	42*	AY532402	filamentous	0	0	3	9	0
Penicillium chrysogenum	Ascomycota	saprobe	44*	AB479305	filamentous	0	11	0	0	0
Cylindrocarpon pauciseptatum	Ascomycota	saprobe	50*	EF607080	filamentous	8	0	0	0	0
Penicillium commune	Ascomycota	saprobe	51*	EU551203	filamentous	0	1	4	3	0
Penicillium canescens	Ascomycota	saprobe	52*	FJ230999	filamentous	0	0	1	0	7
Mollisia cinerea	Ascomycota	saprobe	56*	AY259135	filamentous	0	2	4	1	0
Leohumicola atra	Ascomycota	saprobe	57*	EU678386	filamentous	0	0	7	0	0
Corynascus similis	Ascomycota	saprobe	59*	AJ224201	filamentous	0	0	6	0	1
Corynascus verrucosus	Ascomycota	saprobe	62*	AJ224203	filamentous	0	1	1	5	0
Geomyces pannorum var. *asperulatus*	Ascomycota	saprobe	65*	AJ390390	filamentous	0	0	0	0	6
Paecilomyces carneus	Ascomycota	saprobe	67*	FN394726	filamentous	0	0	0	0	6
Epicoccum nigrum	Ascomycota	saprobe	73*	GQ996573	filamentous	1	3	2	0	0
Capnobotryella sp. nov. NH43	Ascomycota	saprobe	86	AJ301706	filamentous	0	0	0	4	0
Aureobasidium pullulans	Ascomycota	saprobe	93	AY141180	dimorphic	0	0	0	4	0
Myrothecium verrucaria	Ascomycota	saprobe	99*	AJ301999	filamentous	0	3	0	0	0
Capnobotryella sp. nov. NH43	Ascomycota	saprobe	102	AJ301706	filamentous	0	1	1	1	0
Penicillium purpurogenum	Ascomycota	saprobe	104	DQ681328	filamentous	0	0	0	2	1
Sporothrix inflata	Ascomycota	saprobe	107	AY495429	dimorphic	0	0	1	1	1
Antarctomyces psychrotrophicus	Ascomycota	saprobe	109	FJ911878	filamentous	0	0	0	3	0
Phialocephala xalapensis	Ascomycota	saprobe	110	AF486128	filamentous	0	0	0	0	3
Hyalodendriella betulae	Ascomycota	saprobe	114*	GQ241281	filamentous	0	3	0	0	0
Chrysosporium pseudomerdarium	Ascomycota	saprobe	117	EU823311	filamentous	0	1	2	0	0
Chaetosphaeria chloroconia	Ascomycota	saprobe	119*	AF178542	filamentous	0	2	0	0	0
Eupenicillium levitum	Ascomycota	saprobe	121	AF033436	filamentous	0	0	0	0	2
Leotia lubrica	Ascomycota	saprobe	123	AY144547	filamentous	0	0	0	0	2
Aporospora terricola	Ascomycota	saprobe	125*	DQ865097	filamentous	2	0	0	0	0
Acremonium strictum	Ascomycota	saprobe	126	DQ132831	filamentous	0	0	2	0	0
Sarcinomyces petricola	Ascomycota	saprobe	134*	AJ244274	dimorphic	0	2	0	0	0
Spiromastix warcupii	Ascomycota	saprobe	135*	AY527407	filamentous	1	0	1	0	0
Corynascus verrucosus	Ascomycota	saprobe	141	FJ537093	filamentous	0	0	0	2	0
Myxotrichum deflexum	Ascomycota	saprobe	142*	AF062814	filamentous	1	0	0	1	0
Penicillium lividum	Ascomycota	saprobe	146	AY373922	filamentous	0	0	0	0	2
Periconia macrospinosa	Ascomycota	saprobe	147*	FJ536207	filamentous	1	0	1	0	0
Phialophora lignicola	Ascomycota	saprobe	148	AF083193	filamentous	0	0	2	0	0
Chalara microchona	Ascomycota	saprobe	151*	AY590782	filamentous	0	2	0	0	0
Trichocladium opacum	Ascomycota	saprobe	152	GQ179993	filamentous	0	0	0	0	2
Penicillium megasporum	Ascomycota	saprobe	153*	AF033494	filamentous	0	2	0	0	0
Cochliobolus dactyloctenii	Ascomycota	saprobe	155*	AF071322	filamentous	2	0	0	0	0
Penicillium roseopurpureum	Ascomycota	saprobe	156	FJ231018	filamentous	0	0	0	0	2
Articulospora proliferata	Ascomycota	saprobe/aquatic	81*	FJ000395	filamentous	0	3	0	2	0
Articulospora proliferata	Ascomycota	saprobe/aquatic	90*	FJ000395	filamentous	1	0	3	0	0
Tetracladium marchalianum	Ascomycota	saprobe/aquatic	97*	AF411024	filamentous	2	1	0	0	0
Zalerion varium	Ascomycota	saprobe/aquatic	112	AJ608987	filamentous	0	0	2	1	0
Articulospora proliferata	Ascomycota	saprobe/aquatic	132*	FJ000395	filamentous	0	2	0	0	0
Phialocephala fluminis	Ascomycota	saprobe/aquatic	133*	AF486124	filamentous	0	2	0	0	0
Articulospora proliferata	Ascomycota	saprobe/aquatic	143	FJ000395	filamentous	0	0	2	0	0
Trichoderma viride	Ascomycota	saprobe/mycoparasite/zoopathogen	4*	GU067751	filamentous	0	0	13	56	30
Fusarium brachygibbosum	Ascomycota	saprobe/phytoparasite	34*	GQ505450	filamentous	5	3	9	0	0
Fusarium lateritium	Ascomycota	saprobe/phytoparasite	63*	AB470895	filamentous	0	0	0	0	7
Discostroma tricellulare	Ascomycota	saprobe/phytoparasite	70*	EU030327	filamentous	0	0	6	0	0
Poculum henningsianum	Ascomycota	saprobe/phytoparasite	79*	U21820	filamentous	0	5	0	0	0
Verticillium nigrescens	Ascomycota	saprobe/phytoparasite	87*	FN386267	filamentous	1	0	0	3	0
Massarina rubi	Ascomycota	saprobe/phytoparasite	118*	AF383963	filamentous	2	1	0	0	0
Discostroma tricellulare	Ascomycota	saprobe/phytoparasite	130*	EU030327	filamentous	0	2	0	0	0
Helgardia anguioides	Ascomycota	saprobe/phytoparasite/endophyte	77*	AY266144	filamentous	0	0	4	1	0
Cladosporium cladosporioides	Ascomycota	saprobe/zoopathogen	9*	GQ458030	filamentous	2	2	13	27	0
Alternaria alternata	Ascomycota	saprobe/zoopathogen	82*	GU062279	filamentous	1	2	2	0	0
Lecythophora hoffmannii	Ascomycota	saprobe/zoopathogen	105	FJ903377	filamentous	0	0	0	3	0
Lewia infectoria	Ascomycota	saprobe/zoopathogen	131	FJ433875	filamentous	0	0	0	2	0
Cortinarius gallurae	Basidiomycota	ectomycorrhizic	19*	FN428982	filamentous	0	0	0	7	19
Gymnomyces subfulvus	Basidiomycota	ectomycorrhizic	28*	AY239322	filamentous	0	0	0	0	19
Cortinarius trivialis	Basidiomycota	ectomycorrhizic	41*	DQ295112	filamentous	0	0	0	0	12
Cortinarius incisus	Basidiomycota	ectomycorrhizic	60*	AY669656	filamentous	0	0	0	0	7
Cortinarius rubricosus	Basidiomycota	ectomycorrhizic	66	AY669673	filamentous	0	0	0	4	2
Cortinarius fasciatus	Basidiomycota	ectomycorrhizic	96	GQ159913	filamentous	0	0	0	0	3
Russula aeruginea	Basidiomycota	ectomycorrhizic	6*	UDB001621	filamentous	0	0	0	41	7

Russula odorata	Basidiomycota	ectomycorrhizic	10*	AY061698	filamentous	0	0	0	23	14
Cortinarius casimiri	Basidiomycota	ectomycorrhizic	13*	GQ159893	filamentous	0	0	0	5	26
Inocybe asterospora	Basidiomycota	ectomycorrhizic	18*	UDB000098	filamentous	0	0	0	0	26
Russula virescens	Basidiomycota	ectomycorrhizic	22*	UDB000117	filamentous	0	0	0	0	24
Russula vinosa	Basidiomycota	ectomycorrhizic	29*	UDB002426	filamentous	0	0	0	0	18
Russula abietina	Basidiomycota	ectomycorrhizic	36*	EU598179	filamentous	0	0	0	0	14
Amanita phalloides	Basidiomycota	ectomycorrhizic	37*	UDB002182	filamentous	0	0	0	0	13
Russula cyanoxantha	Basidiomycota	ectomycorrhizic	49*	UDB001483	filamentous	0	0	0	0	9
Russula pectinatoides	Basidiomycota	ectomycorrhizic	61*	AY880930	filamentous	0	0	0	7	0
Tomentella sublilacina	Basidiomycota	ectomycorrhizic	68*	UDB003301	filamentous	0	0	0	6	0
Scleroderma verrucosum	Basidiomycota	ectomycorrhizic	69*	AJ629886	filamentous	0	0	0	6	0
Lactarius serifluus	Basidiomycota	ectomycorrhizic	72*	UDB000868	filamentous	0	0	0	0	6
Sebacina helvelloides	Basidiomycota	ectomycorrhizic	74*	UDB000972	filamentous	0	0	0	0	6
Laccaria amethystina	Basidiomycota	ectomycorrhizic	76*	AM087255	filamentous	0	0	0	5	0
Geastrum minimum	Basidiomycota	ectomycorrhizic	78*	EU784237	filamentous	0	0	0	5	0
Boletus calopus	Basidiomycota	ectomycorrhizic	88	AJ296293	filamentous	0	0	0	4	0
Entoloma prunuloides	Basidiomycota	ectomycorrhizic	94	DQ206983	filamentous	0	0	0	4	0
Tomentella stuposa	Basidiomycota	ectomycorrhizic	101	EU819523	filamentous	0	0	0	3	0
Thelephora penicillata	Basidiomycota	ectomycorrhizic	103	U83484	filamentous	0	0	0	0	3
Clavulina cinerea	Basidiomycota	ectomycorrhizic	128	EU118616	filamentous	0	0	0	0	2
Hymenogaster tener	Basidiomycota	ectomycorrhizic	137	EU784364	filamentous	0	0	0	0	2
Thelephora anthocephala	Basidiomycota	ectomycorrhizic	138	DQ974771	filamentous	0	0	0	0	2
Russula xerampelina	Basidiomycota	ectomycorrhizic	149	FJ845433	filamentous	0	0	0	0	2
Gyroporus castaneus	Basidiomycota	ectomycorrhizic	150	UDB000651	filamentous	0	0	0	0	2
Cortinarius stillatitius	Basidiomycota	ectomycorrhizic	3*	UDB001569	filamentous	0	0	0	0	129
Hygrophorus persoonii	Basidiomycota	ectomycorrhizic[3]	7*	UDB001191	filamentous	0	0	0	0	48
Ceratobasidium cornigerum	Basidiomycota	orchid endophyte	85*	EU273525	filamentous	2	0	0	1	1
Rhizoctonia solani	Basidiomycota	phytoparasite/orchid endophyte	30*	EU730859	filamentous	0	0	18	0	0
Cryptococcus phenolicus	Basidiomycota	saprobe	2*	AF444351	yeast-like	4	39	100	44	10
Cryptococcus victoriae	Basidiomycota	saprobe	16*	AJ581047	yeast-like	0	0	8	17	2
Cryptococcus aerius	Basidiomycota	saprobe	20*	AF145324	yeast-like	4	1	15	5	0
Cryptococcus macerans	Basidiomycota	saprobe	53*	EU082230	yeast-like	0	1	1	6	0
Marasmiellus paspali	Basidiomycota	saprobe	54*	EF175511	filamentous	0	0	1	7	0
Cryptococcus antarcticus	Basidiomycota	saprobe	71*	AB032670	yeast-like	0	6	0	0	0
Coprinellus radians	Basidiomycota	saprobe	98*	AY461815	filamentous	3	0	0	0	0
Lycoperdon perlatum	Basidiomycota	saprobe	111	EU622257	filamentous	0	0	2	1	0
Sporobolomyces nylandii	Basidiomycota	saprobe	122*	AB030323	yeast-like	0	2	0	0	0
Cystofilobasidium infirmominiatum	Basidiomycota	saprobe	144*	AY264716	yeast-like	0	2	0	0	0
Uthatobasidium fusisporum	Basidiomycota	saprobe/orchid endophyte	108*	DQ398957	filamentous	1	0	0	2	0
Hygrocybe psittacina var. *Psittacina*[5]	Basidiomycota	saprobe[3]	15*	FM208875	filamentous	0	0	0	0	28
Olpidium brassicae	Chytridiomycota	phytoparasite	113*	AB205207	holocarpic thallus	0	3	0	0	0
Paraglomus laccatum	Glomeromycota	endomycorrhizic	124	AM295494	filamentous	0	0	0	2	0
Mortierella alpina	Zygomycota	saprobe	27*	EF519894	filamentous	6	0	1	0	13
Mortierella elongata	Zygomycota	saprobe	145*	AJ878504	filamentous	0	2	0	0	0
Mortierella exigua	Zygomycota	saprobe	39*	FJ161926	filamentous	6	6	1	0	0
Mortierella gamsii	Zygomycota	saprobe	84*	EF152527	filamentous	1	2	0	1	0
Mortierella minutissima	Zygomycota	saprobe	38*	AB476417	filamentous	0	1	0	0	12
Umbelopsis isabellina	Zygomycota	saprobe	55*	GU062258	filamentous	0	0	0	0	7
Umbelopsis ramanniana	Zygomycota	saprobe	26*	DQ888724	filamentous	0	11	3	6	0

For each OTU is reported: the phylum of origin, the putative fungal lifestyle, the OTU identification number, the closest accession number obtained against the reference databases (UNITE and GenBank), the habit on the basis of the identification, and the number of sequences retrieved in each soil. TV, tilled vineyard; CV, covered vineyard; MM, managed meadow; PA, pasture; CO, cork-oak formation.

* = OTUs considered for the statistical analysis due to their abundance (>1% in at least one soil).

[1] Tedersoo L, May TW, Smith ME (2010) Ectomycorrhizal lifestyle in fungi: global diversity, distribution, and evolution of phylogenetic lineages. Mycorrhiza 20: 217-263.

[2] Doveri, F (2004) Fungi Fimicoli Italici. Trento, Italy: Associazione Micologica Bresadola Fondazione Centro Studi Micologici dell'A.M.B.

[3] Seitzman BH, Ouimette A, Mixon RL, Hobbie EA, Hibbett DS (2011) Conservation of biotrophy in Hygrophoraceae inferred from combined stable isotope and phylogenetic analyses. Mycologia 103: 280-290.

[4] Genney DR, Hale AD, Woods RG, Wright M (2009) Grassland fungi. In: Guidelines for selection of biological SSSIs Rationale Operational approach and criteria Detailed guidelines for habitats and species groups. UK: Joint Nature Conservation Committee.

[5] Griffith GW, Easton GL, Jones AW (2002) Ecology and Diversity of Waxcap (/Hygrocybe/ spp.) Fungi. Bot J Scotl 54: 7-22.
