# Supplementary material for: Unravelling Soil Fungal Communities from Different Mediterranean Land-Use Backgrounds
Source: PLoS One. 2012 Apr 20;7(4):e34847. doi: 10.1371/journal.pone.0034847 (PMC3335027; doi:10.1371/journal.pone.0034847)
Supplement: Table S2 — ITS2 OTU abundance in terms of sequence number in the five Sardinian soils. (PDF) [file pone.0034847.s002.pdf]

Table S2

	Phylum	Putative lifestyle[1]	OTU number	Closest accession number	Habit	CV (no. seq.)	TV	MM	PA	CO
Podospora miniglutinans	Ascomycota	coprophile[2]	64	AY515362	filamentous	0	4	2	2	0
Podospora tetraspora	Ascomycota	coprophile	68	GQ922572	filamentous	0	0	5	2	0
Podospora inflatula	Ascomycota	coprophile	72	GQ922537	filamentous	0	1	5	0	0
Podospora intestinacea	Ascomycota	coprophile	117	AY515363	filamentous	0	2	0	0	0
Ascobolus immersus	Ascomycota	coprophile	148	AJ271628	filamentous	0	0	0	2	0
Ascobolus crenulatus	Ascomycota	coprophile	167	DQ491504	filamentous	0	0	0	2	0
Thelebolus microsporus	Ascomycota	coprophile	25	GQ483644	filamentous	0	0	13	28	0
Cenococcum geophilum	Ascomycota	ectomycorrhizic[1]	41	DQ068980	filamentous	0	0	0	0	14
Cenococcum geophilum	Ascomycota	ectomycorrhizic	45	AB089815	filamentous	1	2	7	2	1
Humaria hemisphaerica	Ascomycota	ectomycorrhizic	113	UDB000988	filamentous	0	0	0	0	3
Metarhizium anisopliae	Ascomycota	entomopathogen	47	FJ609304	filamentous	5	6	0	0	0
Oidiodendron flavum	Ascomycota	ericoid endophye/ectomycorrhizic	23	AF307763	filamentous	0	0	1	0	45
Oidiodendron chlamydosporicum	Ascomycota	ericoid endophye/ectomycorrhizic	102	AF062789	filamentous	0	0	0	0	4
Oidiodendron pilicola	Ascomycota	ericoid endophye/ectomycorrhizic	131	AF062787	filamentous	0	0	0	0	2
Oidiodendron maius	Ascomycota	ericoid endophye/ectomycorrhizic	169	AF307771	filamentous	0	0	2	0	0
Hypocrea lixii	Ascomycota	mycoparasite	74	FJ884157	filamentous	0	0	0	0	6
Leptodontidium orchidicola	Ascomycota	orchid endophyte	27	AF214576	filamentous	2	13	13	2	0
Fusarium oxysporum	Ascomycota	phytoparasite	8	GQ922563	filamentous	0	3	7	10	0
Fusarium oxysporum	Ascomycota	phytoparasite	9	GQ922563	filamentous	13	44	127	231	19
Fusarium equiseti	Ascomycota	phytoparasite	38	GQ505681	filamentous	8	2	4	1	0
Fusarium tricinctum	Ascomycota	phytoparasite	40	GQ922561	filamentous	0	4	7	2	1
Drechslera avenae	Ascomycota	phytoparasite	44	AF260328	filamentous	0	0	13	0	0
Drechslera fugax	Ascomycota	phytoparasite	62	AY004791	filamentous	0	0	8	0	0
Plectosphaerella cucumerina	Ascomycota	phytoparasite	63	FM178318	filamentous	0	1	4	3	0
Phoma samarorum	Ascomycota	phytoparasite	84	FJ427062	filamentous	0	0	5	0	0
Sclerotinia minor	Ascomycota	phytoparasite	85	AB516661	filamentous	0	5	0	0	0
Pyrenochaeta lycopersici	Ascomycota	phytoparasite	96	DQ865111	filamentous	0	1	3	0	0
Mycoleptodiscus terrestris	Ascomycota	phytoparasite	100	EU364807	filamentous	0	4	0	0	0
Ascochyta skagwayensis	Ascomycota	phytoparasite	128	AF520640	filamentous	0	0	0	3	0
Neonectria radicicola	Ascomycota	phytoparasite	129	AY295311	filamentous	0	0	2	0	1
Phomopsis columnaris	Ascomycota	phytoparasite	130	FN394688	filamentous	0	0	2	0	0
Veronaea musae	Ascomycota	phytoparasite	134	GQ184730	filamentous	1	1	0	0	0
Truncatella angustata	Ascomycota	phytoparasite	143	GU062256	filamentous	2	0	0	0	0
Sclerotinia spermophila	Ascomycota	phytoparasite	146	AJ716305	filamentous	0	0	2	0	0
Neonectria trachosa	Ascomycota	phytoparasite	149	AY677297	filamentous	0	0	1	1	0
Venturia hystrioides	Ascomycota	phytoparasite	159	EU035459	filamentous	0	2	0	0	0
Verticillium dahliae	Ascomycota	phytoparasite	163	DQ282123	filamentous	0	2	0	0	0
Phaeosphaeria oreochloae	Ascomycota	phytoparasite/endophyte	104	AF439494	filamentous	0	0	3	0	0
Fusarium solani	Ascomycota	phytoparasite/zoopathogen	82	GQ922912	filamentous	2	0	3	0	0
Torula herbarum	Ascomycota	saprobe	1	FJ946483	filamentous	0	0	0	0	3
Torula herbarum	Ascomycota	saprobe	2	FJ946483	filamentous	0	0	0	1	1
Thielavia terricola	Ascomycota	saprobe	3	AJ271579	filamentous	1	4	38	14	4
Chaetomium globosum	Ascomycota	saprobe	4	AB511970	filamentous	14	61	118	429	11
Torula herbarum	Ascomycota	saprobe	5	FJ946483	filamentous	2	8	3	32	60
Penicillium janczewskii	Ascomycota	saprobe	13	FJ230987	filamentous	1	0	1	8	23
Penicillium restrictum	Ascomycota	saprobe	14	AF033457	filamentous	2	5	1	3	29
Trichoderma gamsii	Ascomycota	saprobe	15	GQ922545	filamentous	0	0	2	55	12
Epicoccum nigrum	Ascomycota	saprobe	19	GQ996573	filamentous	5	0	13	33	1
Penicillium urticae	Ascomycota	saprobe	21	GQ389620	filamentous	0	0	0	12	31
Neosartorya stramenia	Ascomycota	saprobe	22	AF459733	filamentous	0	0	1	3	0
Microglossum olivaceum	Ascomycota	saprobe	24	EU784373	filamentous	0	0	0	0	43
Ulocladium tuberculatum	Ascomycota	saprobe	28	FJ914692	filamentous	1	7	15	3	0
Aureobasidium pullulans	Ascomycota	saprobe	29	AY141180	dimorphic	0	2	7	12	4
Spiromastix tentaculatum	Ascomycota	saprobe	33	AY527406	filamentous	0	8	6	7	0
Geomyces pannorum var. *asperulatus*	Ascomycota	saprobe	35	AJ390390	filamentous	0	0	1	2	14
Chrysosporium pseudomerdarium	Ascomycota	saprobe	36	EU823311	filamentous	0	2	5	8	1
Chaetomium globosum	Ascomycota	saprobe	39	FJ791145	filamentous	0	0	2	13	0
Rhizosphaera macrospora	Ascomycota	saprobe	42	AM884745	filamentous	3	11	0	0	0
Chaetomium funicola	Ascomycota	saprobe	46	GQ996574	filamentous	0	0	1	11	0
Penicillium simplicissimum	Ascomycota	saprobe	54	AF203084	filamentous	0	2	0	7	0
Preussia aemulans	Ascomycota	saprobe	57	AY943044	filamentous	0	4	0	5	0
Corynascus sexualis	Ascomycota	saprobe	66	AJ224202	filamentous	0	6	1	0	0
Geoglossum glutinosum[3]	Ascomycota	saprobe	69	EU784443	filamentous	0	0	0	0	7
Pyrenopeziza revincta	Ascomycota	saprobe	73	AJ430224	filamentous	0	2	4	0	0
Penicillium radicum	Ascomycota	saprobe	75	EU262660	filamentous	0	1	0	5	0
Penicillium pinophilum	Ascomycota	saprobe	77	GQ422445	filamentous	0	0	0	6	0
Phialocephala xalapensis	Ascomycota	saprobe	80	AF486128	filamentous	0	4	1	0	0
Paecilomyces carneus	Ascomycota	saprobe	83	FN394726	filamentous	0	0	0	2	3
Penicillium purpurogenum	Ascomycota	saprobe	88	DQ681328	filamentous	0	0	0	2	2
Pseudeurotium bakeri	Ascomycota	saprobe	95	FJ903285	filamentous	0	0	1	3	0
Lophiostoma fuckelii	Ascomycota	saprobe	97	EU552139	filamentous	0	0	0	4	0
Cephalotheca sulfurea	Ascomycota	saprobe	99	EU823315	filamentous	0	0	3	1	0
Trichoderma tomentosum	Ascomycota	saprobe	105	FJ861465	filamentous	0	0	0	0	3
Trichoderma hamatum	Ascomycota	saprobe	110	GQ220703	filamentous	0	0	0	0	3
Cistella grevillei	Ascomycota	saprobe	111	U57089	filamentous	0	0	3	0	0
Veronaeopsis simplex	Ascomycota	saprobe	112	EU041820	filamentous	0	3	0	0	0
Spiromastix tentaculatum	Ascomycota	saprobe	114	AY527406	filamentous	0	0	1	2	0
Geoglossum nigritum	Ascomycota	saprobe	116	DQ491490	filamentous	0	0	0	0	3
Talaromyces rotundus	Ascomycota	saprobe	118	AF285115	filamentous	0	0	0	3	0
Amorphotheca resinae	Ascomycota	saprobe	122	EU030275	filamentous	2	0	1	0	0
Chaetosphaeria inaequalis	Ascomycota	saprobe	124	AF178564	filamentous	0	0	0	0	3
Aspergillus niger	Ascomycota	saprobe	125	GU082483	filamentous	0	3	0	0	0
Pseudogymnoascus roseus	Ascomycota	saprobe	126	AY608922	filamentous	0	0	1	2	0
Trichocladium opacum	Ascomycota	saprobe	133	GQ179993	filamentous	0	0	0	0	2
Penicillium janczewski	Ascomycota	saprobe	135	FJ861429	filamentous	0	0	0	0	2
Cistella grevillei	Ascomycota	saprobe	137	U57089	filamentous	0	0	2	0	0
Trichocladium pyriforme	Ascomycota	saprobe	140	AM292048	filamentous	0	0	0	2	0
Penicillium pimiteouiense	Ascomycota	saprobe	150	AF037431	filamentous	0	1	1	0	0
Penicillium megasporum	Ascomycota	saprobe	152	AF033494	filamentous	1	1	0	0	0
Amorphotheca resinae	Ascomycota	saprobe	153	EU030275	filamentous	0	0	0	2	0
Sporothrix inflata	Ascomycota	saprobe	155	AY495425	filamentous	0	0	2	0	0
Lophiostoma cynaroidis	Ascomycota	saprobe	156	EU552138	filamentous	0	0	0	0	2
Lipomyces starkeyi	Ascomycota	saprobe	157	U82459	yeast-like	2	0	0	0	0
Acremonium strictum	Ascomycota	saprobe	160	FM998714	filamentous	0	0	2	0	0
Merimbla humicoloides	Ascomycota	saprobe	164	AF368298	filamentous	0	0	0	0	2
Penicillium brasilianum	Ascomycota	saprobe	166	AB455514	filamentous	0	0	0	2	0
Phialocephala fluminis	Ascomycota	saprobe/aquatic	26	AF486124	filamentous	0	17	7	7	0
Articulospora proliferata	Ascomycota	saprobe/aquatic	51	FJ000395	filamentous	0	1	3	6	0
Zalerion varium	Ascomycota	saprobe/aquatic	71	AF169303	filamentous	0	0	3	3	0
Tetracladium furcatum	Ascomycota	saprobe/aquatic	90	AF411026	filamentous	0	0	4	0	0
Phialocephala fluminis	Ascomycota	saprobe/aquatic	94	AF486124	filamentous	0	3	0	1	0
Neurospora africana	Ascomycota	saprobe/coprophile	91	GQ922531	filamentous	1	0	3	0	0
Trichoderma spirale	Ascomycota	saprobe/endophyte	60	EF596946	filamentous	0	1	0	0	7
Mycosphaerella verrucosiafricana	Ascomycota	saprobe/phytoparasite	37	EU301087	filamentous	0	0	0	16	0
Poculum henningsianum	Ascomycota	saprobe/phytoparasite	48	Z81442	filamentous	0	8	1	2	0
Discostroma fuscellum	Ascomycota	saprobe/phytoparasite	50	AF377284	filamentous	0	0	9	0	1
Fusarium brachygibbosum	Ascomycota	saprobe/phytoparasite	55	GQ505450	filamentous	0	3	6	0	0
Cylindrocarpon pauciseptatum	Ascomycota	saprobe/phytoparasite	79	EF607080	filamentous	5	0	0	0	0

Poculum henningsianum	Ascomycota	saprobe/phytoparasite	86	Z81442	filamentous	0	2	0	2	1
Montagnula opulenta	Ascomycota	saprobe/phytoparasite	144	AF383966	filamentous	2	0	0	0	0
Cladosporium cladosporioides	Ascomycota	saprobe/zoopathogen	16	GQ458030	filamentous	1	3	13	48	3
Lecythophora hoffmannii	Ascomycota	saprobe/zoopathogen	52	AY945807	filamentous	0	0	0	9	0
Pleurophoma cava	Ascomycota	saprobe/zoopathogen	139	GU062248	filamentous	0	0	0	2	0
Bipolaris spicifera	Ascomycota	saprobe/zoopathogen	151	AF163076	filamentous	2	0	0	0	0
Phaeoannellomyces elegans	Ascomycota	zoopathogen	136	EF551549	dimorphic	0	0	0	2	0
Hygrophorus persoonii[4]	Basidiomycota	ectomycorrhizic	10	UDB001191	filamentous	0	0	3	0	116
Inocybe asterospora	Basidiomycota	ectomycorrhizic	32	UDB000098	filamentous	0	0	0	0	21
Russula aeruginea	Basidiomycota	ectomycorrhizic	43	UDB001621	filamentous	0	0	0	11	3
Gymnomyces subfulvus	Basidiomycota	ectomycorrhizic	49	AY239319	filamentous	0	0	0	0	10
Amanita vaginata	Basidiomycota	ectomycorrhizic	58	UDB002187	filamentous	0	0	0	0	8
Sebacina epigaea	Basidiomycota	ectomycorrhizic	61	UDB000977	filamentous	0	0	0	0	8
Cortinarius trivialis	Basidiomycota	ectomycorrhizic	67	DQ295112	filamentous	0	0	0	0	7
Geastrum minimum	Basidiomycota	ectomycorrhizic	70	EU784237	filamentous	0	0	0	6	0
Russula virescens	Basidiomycota	ectomycorrhizic	78	UDB000117	filamentous	0	0	0	0	6
Laccaria pseudomontana	Basidiomycota	ectomycorrhizic	81	DQ149870	filamentous	0	0	0	5	0
Cortinarius gallurae	Basidiomycota	ectomycorrhizic	87	FN428979	filamentous	0	0	0	5	0
Tomentella sublilacina	Basidiomycota	ectomycorrhizic	89	UDB003301	filamentous	0	0	0	4	0
Geastrum coronatum	Basidiomycota	ectomycorrhizic	98	EU784224	filamentous	0	0	1	3	0
Inocybe cookei	Basidiomycota	ectomycorrhizic	107	AM882953	filamentous	0	0	0	0	3
Russula rubra	Basidiomycota	ectomycorrhizic	109	AY061717	filamentous	0	0	0	0	3
Scleroderma areolatum	Basidiomycota	ectomycorrhizic	115	UDB001212	filamentous	0	0	0	3	0
Gymnomyces californicus	Basidiomycota	ectomycorrhizic	119	AY239318	filamentous	0	0	0	3	0
Russula odorata	Basidiomycota	ectomycorrhizic	120	AY061698	filamentous	0	0	0	0	3
Cortinarius scotoides	Basidiomycota	ectomycorrhizic	121	UDB000167	filamentous	0	0	0	3	0
Geastrum floriforme	Basidiomycota	ectomycorrhizic	145	EU784231	filamentous	0	0	0	2	0
Russula aeruginea	Basidiomycota	ectomycorrhizic	147	UDB001621	filamentous	0	0	0	2	0
Clavulina cristata	Basidiomycota	ectomycorrhizic	154	UDB000073	filamentous	0	0	0	2	0
Russula cyanoxantha	Basidiomycota	ectomycorrhizic	158	UDB000330	filamentous	0	0	0	0	2
Russula sororia	Basidiomycota	ectomycorrhizic	170	AB211275	filamentous	0	0	0	2	0
Cortinarius lividoochraceus	Basidiomycota	ectomycorrhizic	12	UDB001049	filamentous	0	0	0	0	91
Ceratobasidium papillatum	Basidiomycota	orchid endophyte	101	AJ427401	filamentous	0	0	3	1	0
Trechispora alnicola	Basidiomycota	probably ectomycorrhizic	103	DQ411529	filamentous	0	4	0	0	0
Lycoperdon perlatum	Basidiomycota	saprobe	6	EU622257	filamentous	0	0	0	2	0
Lycoperdon perlatum	Basidiomycota	saprobe	7	EU622257	filamentous	0	1	3	7	1
Cryptococcus elinovii	Basidiomycota	saprobe	18	FJ873453	yeast-like	5	21	15	14	7
Cryptococcus podzolicus	Basidiomycota	saprobe	30	FN394715	yeast-like	2	12	0	1	2
Cryptococcus podzolicus	Basidiomycota	saprobe	31	FN394715	yeast-like	0	4	1	2	0
Cryptococcus victoriae	Basidiomycota	saprobe	34	AJ581047	yeast-like	0	0	5	13	0
Cryptococcus laurentii	Basidiomycota	saprobe	65	FJ613122	yeast-like	0	0	2	6	0
Cryptococcus randhawii	Basidiomycota	saprobe	92	AJ876528	yeast-like	0	3	0	1	0
Cryptococcus laurentii	Basidiomycota	saprobe	93	AY315665	yeast-like	0	4	0	0	0
Ceratobasidium albasitensis	Basidiomycota	saprobe	106	AJ427398	filamentous	3	0	0	0	0
Coprinellus radians	Basidiomycota	saprobe	108	AY461815	filamentous	3	0	0	0	0
Cryptococcus podzolicus	Basidiomycota	saprobe	123	FN394715	yeast-like	0	2	1	0	0
Trichosporon porosum	Basidiomycota	saprobe	127	FJ861467	yeast-like	0	0	0	2	1
Cryptococcus chernovii	Basidiomycota	saprobe	142	AF444354	yeast-like	0	0	0	2	0
Cryptococcus watticus	Basidiomycota	saprobe	162	FJ473373	yeast-like	0	2	0	0	0
Psilocybe montana	Basidiomycota	saprobe/coprophile	165	DQ494692	filamentous	0	0	2	0	0
Entrophospora infrequens	Glomeromycota	endomycorrhizic	138	U94714	filamentous	2	0	0	0	0
Mortierella elongata	Zygomycota	saprobe	11	GQ922554	filamentous	26	42	23	9	5
Mortierella elongata	Zygomycota	saprobe	17	GQ922554	filamentous	10	33	10	11	0
Umbelopsis ramanniana	Zygomycota	saprobe	20	DQ888724	filamentous	1	14	14	22	0
Mortierella alpina	Zygomycota	saprobe	53	EF519903	filamentous	4	0	0	0	5
Mortierella minutissima	Zygomycota	saprobe	56	AB476417	filamentous	0	0	1	0	8
Mortierella exigua	Zygomycota	saprobe	59	FJ161926	filamentous	5	1	2	0	0
Mortierella exigua	Zygomycota	saprobe	76	FJ161926	filamentous	4	0	0	0	2
Umbelopsis ramanniana	Zygomycota	saprobe	132	FJ161930	filamentous	0	0	0	0	2
Mortierella alpina	Zygomycota	saprobe	161	EF202181	filamentous	0	2	0	0	0
Rhizopus oryzae	Zygomycota	saprobe/phytoparasite	141	GQ426950	filamentous	0	2	0	0	0
Cunninghamella bertholletiae	Zygomycota	saprobe/zoopathogen	168	AF254931	filamentous	0	2	0	0	0

For each OTU is reported: the phylum of origin, the putative fungal lifestyle, the OTU identification number, the closest accession number obtained against the reference databases (UNITE and GenBank), the habit on the basis of the identification, and the number of sequences retrieved in each soil. Singletons are excluded. TV, tilled vineyard; CV, covered vineyard; MM, managed meadow; PA, pasture; CO, cork-oak formation.

[1] Tedersoo L, May TW, Smith ME (2010) Ectomycorrhizal lifestyle in fungi: global diversity, distribution, and evolution of phylogenetic lineages. Mycorrhiza 20: 217-263.

[2] Doveri, F (2004) Fungi Fimicoli Italici. Trento, Italy: Associazione Micologica Bresadola Fondazione Centro Studi Micologici dell'A.M.B.

[3] Genney DR, Hale AD, Woods RG, Wright M (2009) Grassland fungi. In: Guidelines for selection of biological SSSIs Rationale Operational approach and criteria Detailed guidelines for habitats and species groups. UK: Joint Nature Conservation Committee.

[4] Seitzman BH, Ouimette A, Mixon RL, Hobbie EA, Hibbett DS (2011) Conservation of biotrophy in Hygrophoraceae inferred from combined stable isotope and phylogenetic analyses. Mycologia 103: 280-290.
